# Daily rhythms and enrichment patterns in the transcriptome of the behavior-manipulating parasite *Ophiocordyceps kimflemingiae*

**DOI:** 10.1371/journal.pone.0187170

**Published:** 2017-11-03

**Authors:** Charissa de Bekker, Ian Will, David P. Hughes, Andreas Brachmann, Martha Merrow

**Affiliations:** 1 University of Central Florida, Department of Biology, Orlando, Florida, United States of America; 2 LMU Munich, Institute of Medical Psychology, Faculty of Medicine, Munich, Germany; 3 LMU Munich, Genetics, Faculty of Biology, Planegg-Martinsried, Germany; 4 Pennsylvania State University, Departments of Biology and Entomology, University Park, Pennsylvania, United States of America; McGill University, CANADA

## Abstract

Various parasite-host interactions that involve adaptive manipulation of host behavior display time-of-day synchronization of certain events. One example is the manipulated biting behavior observed in Carpenter ants infected with *Ophiocordyceps unilateralis sensu lato*. We hypothesized that biological clocks play an important role in this and other parasite-host interactions. In order to identify candidate molecular clock components, we used two general strategies: bioinformatics and transcriptional profiling. The bioinformatics approach was used to identify putative homologs of known clock genes. For transcriptional profiling, RNA-Seq was performed on 48 h time courses of *Ophiocordyceps kimflemingiae* (a recently named species of the *O*. *unilateralis* complex), whose genome has recently been sequenced. Fungal blastospores were entrained in liquid media under 24 h light-dark (LD) cycles and were harvested at 4 h intervals either under LD or continuous darkness. Of all *O*. *kimflemingiae* genes, 5.3% had rhythmic mRNAs under these conditions (JTK Cycle, ≤ 0.057 statistical cutoff). Our data further indicates that a significant number of transcription factors have a peaked activity during the light phase (day time). The expression levels of a significant number of secreted enzymes, proteases, toxins and small bioactive compounds peaked during the dark phase or subjective night. These findings support a model whereby this fungal parasite uses its biological clock for phase-specific activity. We further suggest that this may be a general mechanism involved in parasite-host interactions.

## Introduction

Endogenous temporal programs, which anticipate daily changes in e.g. temperature and light, have evolved in organisms from all phyla [1–4]. These programs function as circadian clocks, organizing physiology and behavior to specific times of day (phases). Circadian clocks share several properties, most notably a free-running rhythm of about 24 h in constant conditions and entrainment or synchronization of these rhythms to exactly 24 h in the presence of highly predictable, regular signals from the environment (zeitgebers, e.g. light and temperature cycles). Various insect behaviors, such as foraging, flight and oviposition show phase-characteristic daily rhythms under entrainment conditions [5, 6]. Daily rhythms in immune systems have also been reported in insects, as well as in plants [7, 8], an observation that has consequences for timing of inter-species interactions with respect to parasites. For example, it has been proposed that fungi invade plants such that they circumvent rhythms in host immune systems [9]. Moreover, it has been shown for the plant pathogen *Botrytis cinerea* that its virulence is regulated by the circadian clock [10]. Adaptive timing of infection could thus enable a parasite’s life cycle by increasing infection rates, virulence and, eventually, transmission. In vertebrate-infecting parasites, circadian clocks also appear to play a role. For example, circadian rhythms were shown to contribute to fitness in the malaria parasite *Plasmodium chabaudi* and metabolism in the sleeping sickness parasite *Trypanosoma brucei*, is under clock control [11–13]. Biological clocks could, therefore, be an important aspect of parasite-host interactions in general.

In this work, we wish to learn more about how the circadian clock is used in parasitic behavioral manipulation, specifically in stages past the initial host invasion and immune system evasion. Such a behavioral manipulation can be observed in Formica ants infected with the lancet fluke that are manipulated to bite grass. This behavior appears regulated by daily temperature fluctuations [14–16] and its phase-specific activity facilitates parasite transmission from the intermediate ant host to the gut of the ultimate, herbivore host. Another example of daily synchronization of manipulated behaviors is seen in soldier beetles infected with *Eryniopsis lampyridarum*. Here, infected beetles are thought to grip flower heads with their mandibles in the early morning immediately prior to death. Emergence of conidiophores and conidia (spores; transmission agents) and post-mortem spreading of the wings occurs pre-dawn the following day. This timing is thought to protect the spores via optimal humidity levels [17]. Another time-of-day specific fungal manipulation of insect behavior is that of fungi of the *Ophiocordyceps unilateralis* complex as they infect Carpenter ants. Workers foraging for food are infected when they encounter fungal spores [18]. Infected ants then abandon their tasks as the fungus slowly expands throughout the body [19]. This behavioral change may represent a disruption of the ant circadian clock, as foraging, and other behaviors, are typically under clock control [20, 21]. Ultimately, like in the examples above, the ant displays manipulated biting behavior followed by death at a certain time of day. In field studies in Thailand, *Ophiocordyceps camponoti-leonardi* induced Carpenter ants to bite vegetation around solar noon [22]. Laboratory studies with *Ophiocordyceps kimflemingiae* demonstrated synchronized biting in the early morning [23]. In both *unilateralis* species’ interactions, the timing of death after biting is also synchronous. Moreover, mixed transcriptomics data revealed differentially expressed clock gene homologs in *O*. *kimflemingiae*-infected ants versus healthy individuals sampled at the same time of day [23]. This suggests that the fungal infection works at some level through the host circadian clock.

Fungal clocks have mainly been studied in the nonpathogenic fungus *Neurospora crassa*, revealing a negative feedback loop based on transcriptional regulation that is essential for circadian clock function [24]. In *Neurospora*, the activation arm of this molecular clock gene mechanism consists of the White Collar Complex (WCC). This complex is made up of the transcription factors White Collar-1 (WC-1) and White Collar-2 (WC-2), which have a second role, namely blue-light perception (important for entraining the clock [25–27]) and regulation of light-induced genes (including many clock-controlled genes, ccgs) [28, 29]. As such, the WCC promotes transcription of the *frequency* (*frq*) gene. FRQ forms a complex with FRQ-interacting RNA helicase (FRH), which then functions as the repression arm in the transcriptional feedback loop as it inhibits the activity of WCC. After FRQ is synthesized it is post-translationally modified and degraded. As FRQ levels drop, the WCC is reactivated and the cycle can start anew (reviewed in more detail in e.g. [30–32]). Recent genome-wide gene expression studies in *N*. *crassa*, performed under constant darkness (free-running) conditions, show that the rhythmic expression of the many ccgs peak in time-of-day-related clusters [33, 34]. Catabolic processes appear to be dawn-phased, while anabolic processes are dusk-phased [34]). Additionally, among the circadian clock-regulated genes, processes such as metabolism, protein synthesis, DNA processing and stress response are highly enriched. and phase-specific, with metabolism primarily peaking in the subjective late night and early morning, and protein synthesis activity rising in the subjective afternoon and early night. Expression levels of DNA processing genes also peaked during the subjective late day/evening [33]. Unfortunately, little is known about the molecular mechanism and clock-driven processes in other fungi. *Saccharomyces cerevisiae* shows typical circadian entrainment but no obvious homologs of clock genes are found in its genome [35]. For *Aspergillus* spp. the presence of a circadian clock was also shown, though these ascomycetes have different clock components since no homolog for *frq* was found [36]. Homologs of the *N*. *crassa* “clock genes” have been identified in the basal ascomycete *Pyronema confluens* and rhythmic expression of the *P*.*c*. *frq* has been observed [37]. Furthermore, studies have presented phenotypic and molecular evidence for the presence of a functional molecular clock in the phytopathogenic fungus *B*. *cinerea*, where it appears to contribute to virulence [9, 10]. To a lesser extent, evidence for biological clocks in phytopathogens *Cercospora kikuchii* and *Magnporthe oryzae* have also been reported [38, 39]. To our knowledge, fungal entomopathogens have not yet been studied from a circadian biology perspective.

Circadian rhythms in ants have been reported [20, 21, 40]. To investigate if parasite circadian clocks could contribute to the observed patterns of manipulated ant behavior, we wished to characterize the temporal program in the parasite, *Ophiocordyceps*. To this end, we performed RNA-Seq on two-day long time courses of *O*. *kimflemingiae*. Rhythmic signals were paired with homology searches against *N*. crassa to detect candidate core clock genes in the genome. We report rhythmic candidate clock and clock-controlled genes with activity patterns that appear to be synchronized to certain times of day.

## Materials and methods

### Fungal culture conditions

**Fig 1:**
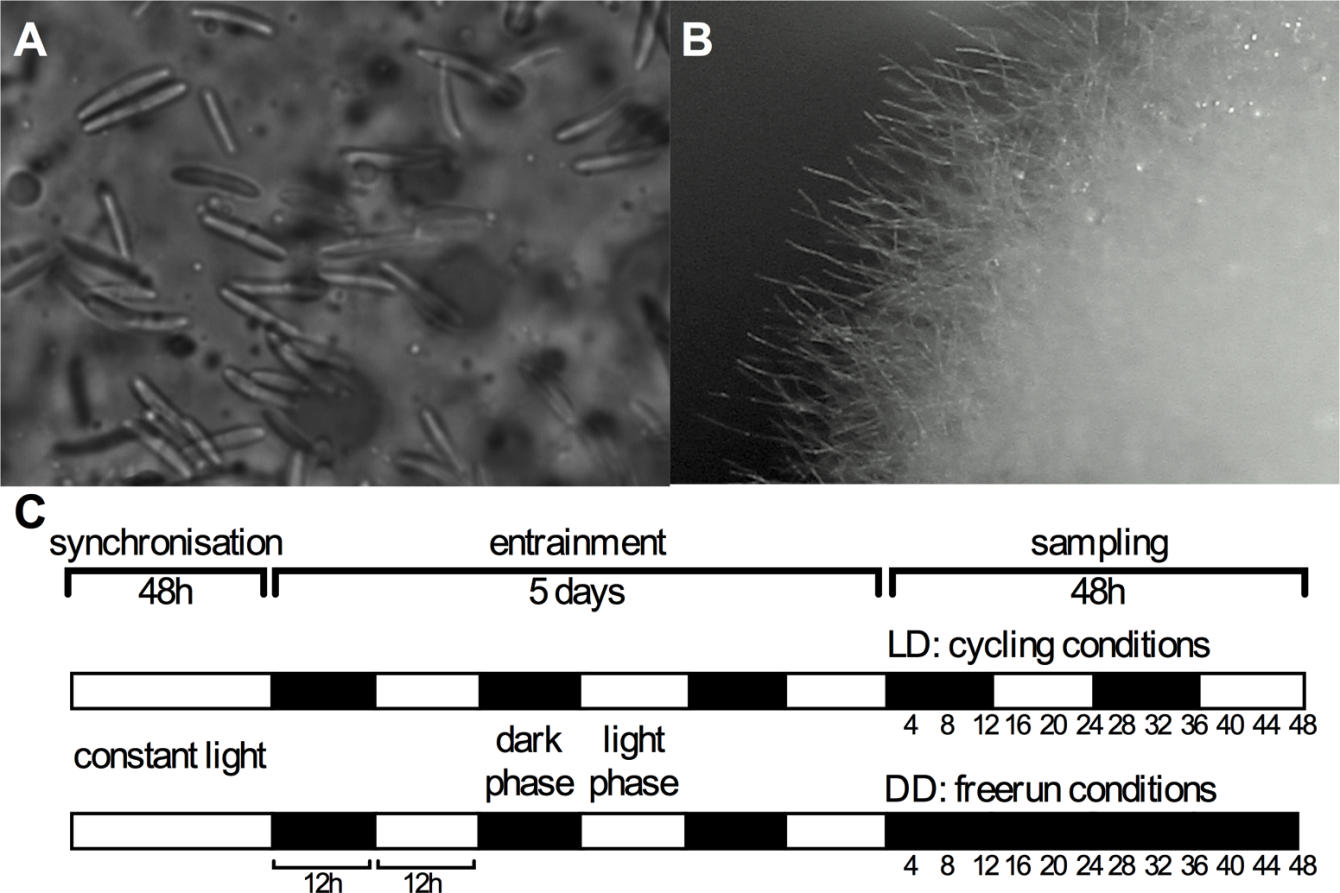
***Ophiocordyceps kimflemingiae* growth conditions** *O*. *kimflemingiae* was grown in its blastospore state (A), rather than in its vegetative growth state (B). Blastospores were synchronized under constant light, followed by entrainment in 24 h LD (12 h:12 h) cycles before harvesting samples under either cycling or free-running conditions (C).

The fungal parasite used in this study, *O*. *kimflemingiae* [41], was isolated from an infected and manipulated *Camponotus castaneus* ant [42]. This species resides within the *O*. *unilateralis s*.*l*. complex and was recently named [41]. Previous data for this species’ interaction with *C*. *castaneus* suggests that biological clocks could be involved in the observed manipulation [23, 42]. Cultures were maintained in Grace’s insect medium (Sigma) supplemented with 10% fetal bovine serum (Invitrogen). For time course studies, cultures were grown as fungal blastospores (Fig 1A) in 25m^2^ tissue culture flasks in 20 mL media and shaken at 60 rpm at 28 ^o^C. Fungal hyphae (Fig 1B) were grown in a 250 mL Erlenmeyer flask holding 100 mL media shaken at the same speed. To synchronize fungal cells, cultures were kept in constant light (light intensity 300 Lux) for 48 h and then transferred to 24 h LD (12 h:12 h) cycles for 5 days at 28 ^o^C. To avoid starvation conditions during sampling, on the fourth day at the end of the light phase, cultures were briefly spun down in a swing-out rotor (3000 rpm for 5 min), and old media was removed and replaced with fresh media. Each culture was subsequently split in two 20 mL cultures: one to keep in LD during sampling, and one to be transferred to DD. After an additional 24 h under LD conditions, half of the cultures were transferred to constant darkness (DD samples). The other half of the flasks were maintained under LD conditions (LD samples) (Fig 1C). During this time, fungal cultures had reached an OD600 of ~ 1 and material was collected every 4 h under red light in a climate controlled room set to 28 ^o^C. Cultures were kept in incubators running zeitgeber cycles in antiphase, which allowed harvesting 48 h time courses over 12 h. Fungal cells were pelleted by spinning down 2 mL of the culture for 3 min at 10,000 rpm. The supernatant was removed and the samples were snap frozen in liquid nitrogen and stored at -80 ^o^C until processing for RNA extraction.

### RNA extraction, library construction and sequencing

Total RNA was extracted as previously reported [43]. Sample quality and quantity were checked with a Bioanalyzer and RNA 6000 Nano Kit (Agilent Technologies), and a Qubit with the RNA BR Assay Kit (Invitrogen). Sequence libraries were constructed using the NEBNext Ultra Directional RNA Library Prep Kit for Illumina (New England BioLabs) and manufacturer’s instructions. Library quality and quantity were checked with a Bioanalyzer and the High Sensitivity DNA Analysis Kit (Agilent Technologies) and a Qubit with the ds DNA HS Assay Kit (Invitrogen). Samples were sequenced on a HiSeq1500 (Illumina) using 50 bp single-end sequencing in rapid-run mode. The dataset is available at the NCBI’s Gene Expression Omnibus under the accession code GSE101312.

### Biostatistical analyses

RNA reads were mapped to the latest version of the *O*. *kimflemingiae* genome [44] with CLC Genomics Workbench 9. Reads were also mapped to inter-genic regions and other parameters were kept at default. Expression levels were calculated for all genes and normalized to Reads Per Kilobase of exon model per Million mapped reads (RPKM).

Cycling transcripts were identified with JTK_CYCLEv3.1 [45]. Normalized expression levels from 4 h-interval time points were used as input data. Defined period length was set at 20–28 hour for the LD time course and at 16–28 hour for the DD time course.

Functional annotation enrichment was determined with a Fisher Exact test with the Benjamini-Hochberg procedure and a corrected p-value cutoff of 0.05 to correct for multiple testing. Hierarchical clustering was performed with Cluster 3.0 and resulting heat maps were visualized with Java TreeView 1.16r4 [46]. For hierarchical clustering, normalized gene expression values were increased by 1 prior to log2-transformation to avoid negative values, and scaled per gene by centering around the mean. Next, genes were hierarchically clustered using the average linkage method.

### Real-time quantitative PCR verification

RNA-Seq results were verified through RT-QPCR by determining the relative expression of five clock gene homologs. Two biological replicates from independently performed time courses and technical triplicates for each time point and amplified gene were used. Primers were designed to span exon-exon boundaries when possible (Table 1). Efficiencies were calculated from a standard curve of serial dilutions. RNA was converted to cDNA with the QuantiTect Reverse Transcription Kit (Qiagen). RT-QPCR reactions were performed on a ViiA 7 (Applied Biosystems) running in Fast Cycling Mode followed by a melt curve analysis, with 10 ng cDNA per 10 μL reaction and PowerUp SYBR Green Master Mix (Applied Biosystems). Amplification data was analyzed using the Relative Quantification and the Design and Analysis Application Apps of the ThermoFisher Cloud Software.

**Table 1:** RT-QPCR primers.

Primer name	Forward Primer (5’-3’)	Reverse Primer (5’-3’)	Amplicon length (bp)	Efficiency (%)
*frq*	ACGACTTGACGGTGGAAATC	TCTTTGCGTAGCATGTCAGG	81	89.58
*wc-1*	AACAGCATGAACCTCGATCC	GAAGAAACGTGGCGAGAAAG	76	94.86
*wc-2*	GAAAAAGGACGGTGCCTATG	AAAGGGTGACTGGTTGTTCG	96	91.17
*vvd*	CTCCAAGGCCGAGATTATTG	ATAGATTGGCGGATCTGCTG	97	96.09
*cry*	TGTCTTGGGAAGAGGGTCAG	TGCGACTTGCGATATGTTGT	169	103.28
2167*	GATGTCATCGATTTCGTACAAGATTC	TGTGCGTCAACATCCTACAGAAG	85	103.34
2393*	GTTCGCGAGGTCTCTGATGAC	GCAGGTTTCCCACATAAACTTATCC	102	90.14
3083*	CGTTCTCAAGGCTCTCAAGG	ACACGGACATTTTGGCCTAC	156	94.46
6497*	ACAACGAGGACCAGCAGAGT	GAACGTCATGACCCTTGAGC	189	102.14
7897*	CTGGGAAGCCTCATCATCGT	GACAACCATGCCCTCTTCGT	75	99.42

Sequences, amplicon length and efficiency of primers used in this study to amplify four clock gene homologs, a putative cryptochrome DASH, and reference genes.

Tested reference genes are indicated with * and their Gene ID numbers are given under primer name. The two most stable reference genes are underlined.

To normalize samples within a time course, candidate reference genes were selected from genes displaying non-cycling expression patterns in the same RNA-Seq dataset, as well as low variation expression patterns in the RNA-Seq dataset from [23]. The pool of non-cycling genes (JTK-CYCLE p >0.057) was first restricted by selecting for genes with a low fold-change, as done in [47] and by setting a cutoff for the coefficient of variation, as described in [48]. Reference candidates displayed a <2-fold-change between the maximum and minimum RPKM expression values under both LD and DD conditions. In addition, log2-transformed expression values displayed a coefficient of variation ≤5%. Reference candidates were further restricted to genes that lacked differential expression in a previously published transcriptomics dataset that compared stages of the fungal parasite in culture, inside the manipulated host and inside the dead host [23]. From this restricted pool, five genes were amplified using RT-QPCR after which the two most stable ones were selected with RefFinder [49] (Table 1).

## Results and discussion

### *O*. *kimflemingiae* blastospores: Growth and sampling conditions

Fungal entomopathogens have a yeast-like (blastospores) and a vegetative (hyphae) growth state. Daily foraging behavior is disrupted and phase-specific biting behavior is induced when the infecting fungus is in a blastospore state (Fig 1A). When secured in the biting position, the host dies and the fungus quickly switches to a vegetative, hyphal growth (Fig 1B). For this study, we identified conditions for *O*. *kimflemingiae* that would induce morphological growth resembling that *in situ* (blastopores in an infected, live *C*. *castaneus* ant).

Culturing *O*. *kimflemingiae* on solid media results in vegetative (hyphal) growth. Hyphae are also formed in liquid shaking cultures grown in Erlenmeyer flasks. In order to trigger blastospore growth in liquid cultures, we tried several media compositions (S1 Table). Vegetative growth persisted in all conditions. We next cultured fungi in tissue culture flasks with rectangular edges shaken gently at 60 rpm. In the presence of Grace’s medium with 10% FBS, the fungi switched to a yeast-like growth and started forming blastospores. Blastospores reverted back to hyphal growth when they were placed into Erlenmeyer flasks. We do not quite understand the reason for this but speculate that this could be due to different aeration rates. The greater turbulence in tissue culture flasks might also decrease flocculation and, therefore, lead to favored blastospore growth. For the experiments in this study, fungal cells were grown in tissue culture flasks throughout time course experiments. Experiments were performed as described in the Materials and methods section and in Fig 1C.

### Mining the *O*. *kimflemingiae* genome for clock gene homologs

Clock and clock-controlled genes of fungi, cyanobacteria, plants, insects and mammals have been reported [1]. We surveyed the *O*. *kimflemingiae* genome for homologs by searching for protein family (Pfam) domains [50] that are encoded in those genes. Per Arnt Sim (PAS) domains (PF00989) are found in all kingdoms of life [51] and have been shown to be involved in eukaryotic circadian clocks [52]. A frequency (FRQ) domain (PF09421) has been identified in the clock gene *frequency* of the fungal model organism *N*. *crassa* [53] as well as other fungi [10, 37]. PHY domains (PF00360) are found in phytochrome photoreceptors that can sense (far-)red light. Phytochromes have been mainly studied in plants where they regulate development via photoperiodism. To a lesser extent, they have been reported for (cyano)bacteria and fungi as well. In *Aspergillus nidulans* this receptor was shown to repress sexual spore formation [54] as it forms a light-regulator complex with receptors similar to the white collar proteins in *N*. *crassa* [55]. In *N*. *crassa*, *however*, *phy* transcripts are not regulated by light. There, *phytochrome-1* mRNAs levels appear under the control of the circadian clock [56]. We mined the *O*. *kimflemingiae* genome for the presence of these functional domains and found eight candidate homolog genes (Table 2). The Pfam search was followed by BlastP alignments against the *N*. *crassa* OR74A genome [57]. These alignments confirmed *O*. *kimflemingiae* homologs of the *N*. *crassa* clock genes *frequency* (*frq)*, *white collar 1* and *2* (*wc-1* and *wc-2*), and *vivid* (*vvd*). In *Neurospora*, VVD is only expressed in the light and regulates gating of light input to the circadian clock [58]. Four additional homologs of PAS domain-containing genes were found through alignment, one of which was a putative *phytochrome 1* (*phy-*1) (Table 2).

**Table 2:** Identified clock(-controlled) gene homologs.

Gene ID	Pfam domains	*Neurospora crassa* OR74A homolog	E value NCBI BlastP	Annotation score UniProt (1–5)
Ophio5|6046	PF09421.5|FRQ	Frequency (*frq*) NCU02265	0.0	5—Experimental evidence
Ophio5|4975	PF13426.1|PAS_9; PF08447.6|PAS_3; PF00989.19|PAS; PF00320.22|GATA	White collar 1 (*wc-1*) NCU02356	0.0	4—Experimental evidence
Ophio5|889	PF00320.22|GATA; PF08447.6|PAS_3; PF13426.1|PAS_9; PF00989.19|PAS	White collar 2 (*wc-2*) NCU00902	2e-174	4—Experimental evidence
Ophio5|6595	PF13426.1|PAS_9	Vivid PAS protein (*vvd*) NCU03967	8e-32	2—Experimental evidence
Ophio5|6786	PF02518.21|HATPase_c; PF00512.20|HisKA; PF00072.19|Response_reg; PF08447.6|PAS_3; PF08448.5|PAS_4	Two-component histidine kinase CHK-1(*nik-2*) NCU01833	0.0	1—Protein predicted
Ophio5|7010	PF02518.21|HATPase_c; PF00072.19|Response_reg; PF00512.20|HisKA; PF13426.1|PAS_9	Development and carotenogenesis control-1 (*dcc-1*) NCU00939	0.0	1—Protein predicted
Ophio5|7293	PF02518.21|HATPase_c; PF00512.20|HisKA; PF00072.19|Response_reg; PF13426.1|PAS_9; PF08447.6|PAS_3; PF08448.5|PAS_4	Autoinducer 2 sensor kinase / phosphatase luxQ NCU02057	0.0	1—Protein predicted
Ophio5|4324	PF00360.15|PHY; PF02518.21|HATPase_c; PF00072.19|Response_reg; PF01590.21|GAF; PF00512.20|HisKA; PF08446.6|PAS_2	Phytochrome-1/Sensor histidine kinase/response regulator (*phy-1*) NCU04834	0.0	1—Protein predicted

Identification of candidate clock(-controlled) genes was based on their functional domains (Pfams). For each identified gene, Pfam domains and the *Neurospora crassa* homolog are given as well as the E value of their alignment and annotation score of the *N*. *crassa* gene according to the UniProt database.

### Detection of candidate genes with cycling transcripts

The identification of clock gene homologs suggests that *O*. *kimflemingiae* may have clock-controlled daily oscillations in gene expression. To detect genes that oscillate under LD and DD conditions, we generated genome wide gene expression profiles of samples collected over a 48 h time course using RNA-Seq. Reads were mapped to the latest version of the *O*. *kimflemingiae* genome, which is 23.92 Mb in size and has been annotated to encode 8,629 genes [44]. Sequencing yielded an average of 20.5 million single-end reads per sample with an average mean quality score of 38. This read depth is around the maximum read depth that was tested for *D*. *melanogaster* [59]. It should thus be sufficient to detect cycling transcripts in our fungal model since its genome is about five times smaller. An average of 95.9% of the reads mapped uniquely to this genome. Resulting expression profiles were normalized to Reads Per Kilobase of exon model per Million mapped reads (RPKM).

Normalized expression profiles were analyzed with the JTK_CYCLE algorithm [45]. Our sampling method (single samples at 4 h intervals) did not allow us to assign false discovery rates. We, therefore, ranked genes based on the Bonferroni-adjusted p-values. In our ranking approach, we relaxed the p = 0.05 threshold, which has been the topic of debate for many years now [60, 61]. We set the threshold for rhythmicity at the p-value of an *Ophiocordyceps* homolog of a rhythmically expressed *Neurospora* clock gene. In *N*. *crassa*, *ca*. 24 h rhythmic expression levels have been observed for *frq* under both LD and DD conditions. Under LD conditions, *vvd* is rhythmic, but when transferred to DD, *vvd* oscillation is only seen in the first 24 h. For *wc-1*, rhythmicity has been observed in LD and though its promoter shows rhythms in DD, its mRNA levels are not predicted to be highly rhythmic [26, 33, 58, 62, 63]. In addition, we used the expression patterns from fungal tissue cultured in LD conditions. JTK_CYCLE returned Bonferroni-adjusted p-values of 0.002, 0.057, and 0.009, respectively, for the above-mentioned homologs under LD conditions. As such, we set our threshold for rhythmicity at the adjusted p-value for *wc-1* under these conditions (i.e. rhythmic candidates p ≤ 0.057). This led to the detection of 333 candidate genes with oscillating transcripts under LD conditions with a median period of 24 h (S1 Fig and S1 Data File). Under DD conditions, adjusted p-values for these genes were markedly higher (0.30, 0.46, and 1 for *frq*, *wc-1*, *vvd*, respectively). This suggests either that endogenous rhythmicity at the mRNA level damps over 48 h in DD and constant temperature conditions or that rhythms detected in LD are simply driven by the zeitgeber cycle. To minimize false discovery, we set the threshold for rhythmicity in DD to the same adjusted p-value as for the LD samples. This led to the detection of 154 genes with suggested circadian rhythms with a median period of 26 h (S1 Fig and S2 Data File). Studies using other model organisms also report fewer rhythmic genes under DD than under LD conditions [64–68]. In addition, lower numbers of cycling transcripts for cells taken out of their natural context and grown under laboratory cell culture conditions have previously been shown [69]. Moreover, a decreased amplitude under DD conditions for the significantly rhythmic gene *frq* is observed in *B*. *cinerea* [10]. Similarly, a decreased amplitude in *O*. kimflemingiae under DD conditions, combined with single 4h-interval data points could have resulted in poor detection of endogenously rhythmic genes in this study.

Among the rhythmic candidate genes in LD, we identified 14 transcription factors (TFs). One of these TFs, as identified above, is the *O*. *kimflemingiae* homolog of *wc-1*. Two of these TFs appear to be involved in regulation of *Neurospora* conidiation (homologs of *N*. *crassa znf-21*; NCU02671 and *csp-2*; NCU06095) and one regulates ascus development (*N*. *crassa* homolog *asd-4*; NCU20921). ChIP sequencing revealed that *N*. *crassa* TFs *znf-21* and *csp-2* are among the direct targets of the White Collar Complex (WCC, formed by WC1 and WC2) [70], a blue-light photoreceptor that is the key transcriptional activator of the circadian oscillator in this fungus. Additionally, we identified a putative blue-light photoreceptor *cryptochrome DASH* homolog (*cry*; NCU00582) (S1 Data File). In *Neurospora*, CRY was shown to be an essential photoreceptor for the correct functioning of a novel *frq*-less-oscillator (FLO) named the CRY-dependent oscillator [71].

We performed enrichment analyses on the rhythmic genes. For both LD and DD conditions, secreted proteins (SPs) and small secreted proteins (SSPs) were significantly over-represented (i.e. LD: 41 SPs and 25 SSPs, Fisher-exact corrected p-value 3.88E-07 and 0.0029, respectively; and DD: 15 SPs and 15 SSPs, Fisher-exact corrected p-value 0.0008 and 0.00160, respectively). One of the SSPs was homologous to *clock-controlled gene-6* of *N*. *crassa* (*ccg-6*; NCU01418). Rhythmic expression has been demonstrated for this conidiation-related gene in *N*. *crassa*, which also appears to be photoinducible and developmentally regulated [72]. Among the rhythmic SPs under LD conditions were five genes encoding enterotoxins and six encoding predicted proteases. These, as well as the potentially bioactive SSPs, could be involved in *O*. *kimflemingiae* interactions with its ant host. Moreover, we found rhythmicity in genes encoding secreted enzymes that have previously been identified as candidate manipulation genes: tyrosinase and protein tyrosine phosphatase, [23].

**Fig 2:**
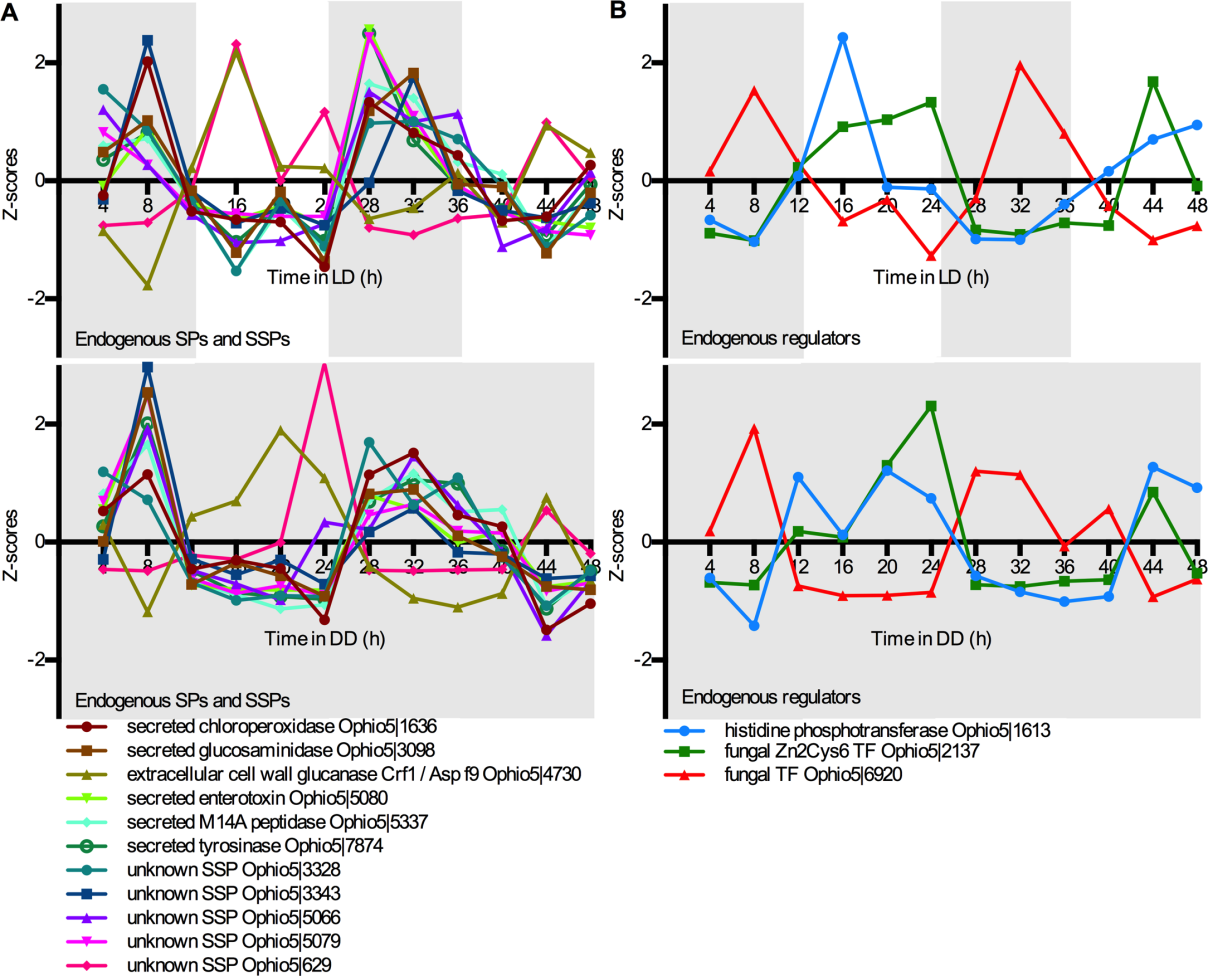
Expression patterns of endogenously cycling genes. (A) Rhythmic mRNA levels of secreted proteins (SPs) and small secreted proteins (SSPs) under cycling (LD) and free-run (DD) conditions. (B) Rhythmic mRNA levels of genes involved in regulatory systems under cycling (LD) and free-run (DD) conditions. Normalized expression levels are plotted as their standard scores (Z-scores) and represent the amplitude of the gene expression observed at that time point versus the mean expression measured over the entire time course of 48 h.

Twenty-six genes had rhythmic transcripts under both cycling and free-running conditions (S1A Fig). A relatively low overlap between LD and DD rhythmic transcripts has previously been observed [65–68, 73]. To obtain insight into the biological processes that were rhythmic regardless of LD or DD conditions, we again performed an enrichment analysis. Despite the small number of endogenously cycling genes (26), we again found an over-representation of SPs (six genes) and SSPs (five genes; Fisher-exact corrected p-value 1.95E-05 and 0.0030, respectively). Nine out of the eleven genes with encoded secretion signals appeared to peak during the dark phase in LD and in the subjective night in DD (Fig 2A). These comprised four unknown SSPs that appear unique to *O*. *kimflemingiae* since they only aligned with deposited sequences of this species in the NCBI Database. Night-active SPs comprised a chloroperoxidase, an exo-beta-D-glucosaminidase, a metallocarboxypeptidase, an enterotoxin, and a tyrosinase. The other two genes peaked during the (anticipated) light phase and comprised another unique SSP with unknown function and a homolog of the *Aspergillus fumigatus* allergen Asp f9, which is a cell wall glucanase (Fig 2A). Two fungal transcription factors (TFs) were also among the endogenously rhythmic genes. We additionally identified a rhythmically expressed histidine phosphotransferase, a gene encoding a predicted two-component regulatory system protein, which would facilitate responses to changing stimuli in the environment [74]. This histidine phosphotransferase, as well as one of the fungal TFs appeared to peak during the (subjective) day, while the other TF showed peaks in expression during the (subjective) night (Fig 2B).

### RT-QPCR verification of rhythmicity

To verify the rhythmic gene expression in our samples, we measured expression of a small subset of genes through RT-QPCR. As target genes, we chose the four clock gene homologs for which experimental evidence in *N*. *crassa* has been reported [63, 75–78]: *frq*, *vvd*, *wc-1* and *wc-2* (Table 2). We added the *O*. *kimflemingiae* homolog for *cry* as a fifth candidate gene (Table 1). All of the genes tested by RT-QPCR were rhythmic in the RNA-Seq data in LD except for *wc-2*, which is constitutively expressed in *Neurospora* [79].

For normalization purposes, we selected candidate reference genes that showed non-rhythmic expression according to the RNA-Seq data. These reference genes were chosen from a pool of 177 genes that did not have cycling transcripts and showed the least expression variations in the RNA-Seq datasets produced in this study and in a previous behavioral manipulation study in *O*. *kimflemingiae* [23] (see Materials and methods section for details). An enrichment analysis using these genes revealed that annotations such as nucleotide, ribonucleoside and ion binding functions, as well as genes with an RNA recognition motif, were over-represented. In addition, (small) secreted proteins were under-represented. After performing RT-QPCR on five of these genes, we used RefFinder [49] to select the two genes that showed the most consistent gene expression across all samples: Ophio5|2167 and Ophio5|2393 (Table 1). Both genes have been functionally predicted to be involved in nucleic acid binding (GO term), and more specifically in RNA binding (Pfam domains). The Genorm score, according to ThermoFisher’s Relative Quantification Cloud Software, for both genes was 0.401.

**Fig 3:**
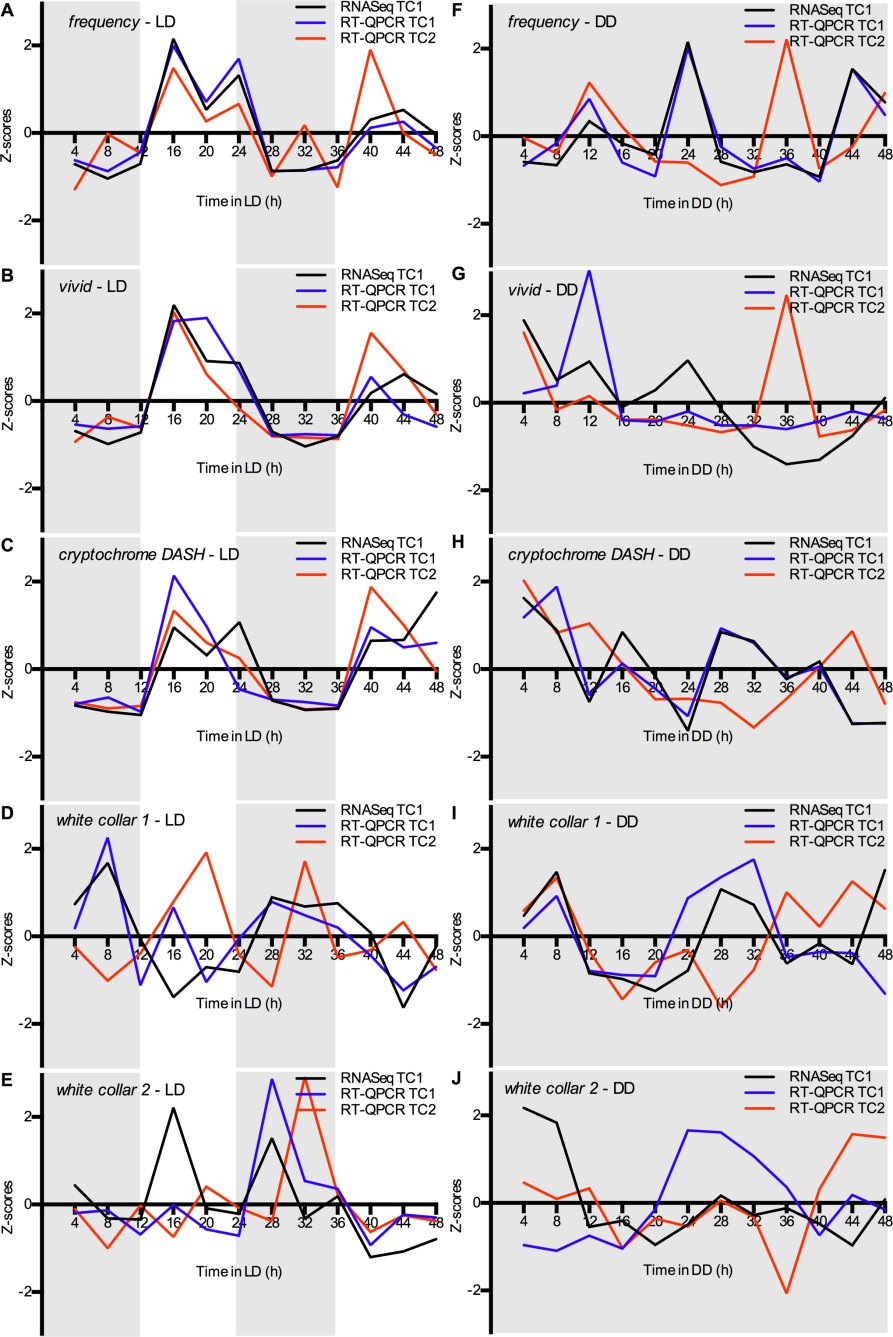
Expression of candidate genes involved in circadian regulation. Expression profiles of four *O*. *kimflemingiae* clock gene homologs and *cryptochrome* over the course of 48 h under LD (A-E) and DD (F-J) conditions. Black lines and blue lines represent RNA-Seq expression levels and RT-QPCR data, respectively, obtained from the same biological replicate (Time Course 1, i.e. TC1). Red lines represent RT-QPCR data obtained from an independently obtained biological replicate (Time Course 2, i.e. TC2). Normalized expression levels are plotted as Z-scores and represent the amplitude of the gene expression observed at that time point versus the mean expression measured over the entire time course of 48 h.

We generated RT-QPCR data from the biological samples that were used to obtain the RNA-Seq dataset (Time Course 1; TC1). In addition, we conducted RT-QPCR on an independently obtained biological replicate for both LD and DD conditions (Time Course 2; TC2). The Z-scores for the normalized RT-QPCR data (RT-QPCR TC1 and RT-QPCR TC2) are plotted together with those for the RNA-Seq profiles (RNASeq TC1) in Fig 3. The means of these three curves and the Standard Error of the Mean (SEM) have been calculated for each time point as well (S2 Fig). Rhythmic genes that were highly expressed (RPKM values) and that displayed a high amplitude (Amp), generally returned lower SEMs. As such, *frq* (average 61.2 RPKM with Amp 35.0), *vvd* (average 73.3 RPKM with Amp 106.4) and *cry* (average 25.0 RPKM with Amp 17.8) under LD conditions (Fig 3 and S2A–S2C Fig) returned average SEMs between 0.19 and 0.22. For *wc-1*, a rhythmic gene with a lower amplitude under LD conditions (average 39.9 RPKM with Amp 8.3), the SEM was higher (average SEM = 0.48; Fig 3 and S2D Fig). The dispersion in the data for this gene, therefore, makes the claim for rhythmicity unwarranted at this time. Expression patterns under DD conditions generally returned higher SEMs as well (average SEMs: 0.33–0.54). The low amplitudes for *frq*, *vvd*, *cry*, and *wc-1* (Amp 2.6–9.3 RPKM) also resulted in expression patterns that displayed more variation across time course experiments (Fig 3 and S2F–S2I Fig).

### Cluster analysis reveals predominantly day- or night-active genes

**Fig 4:**
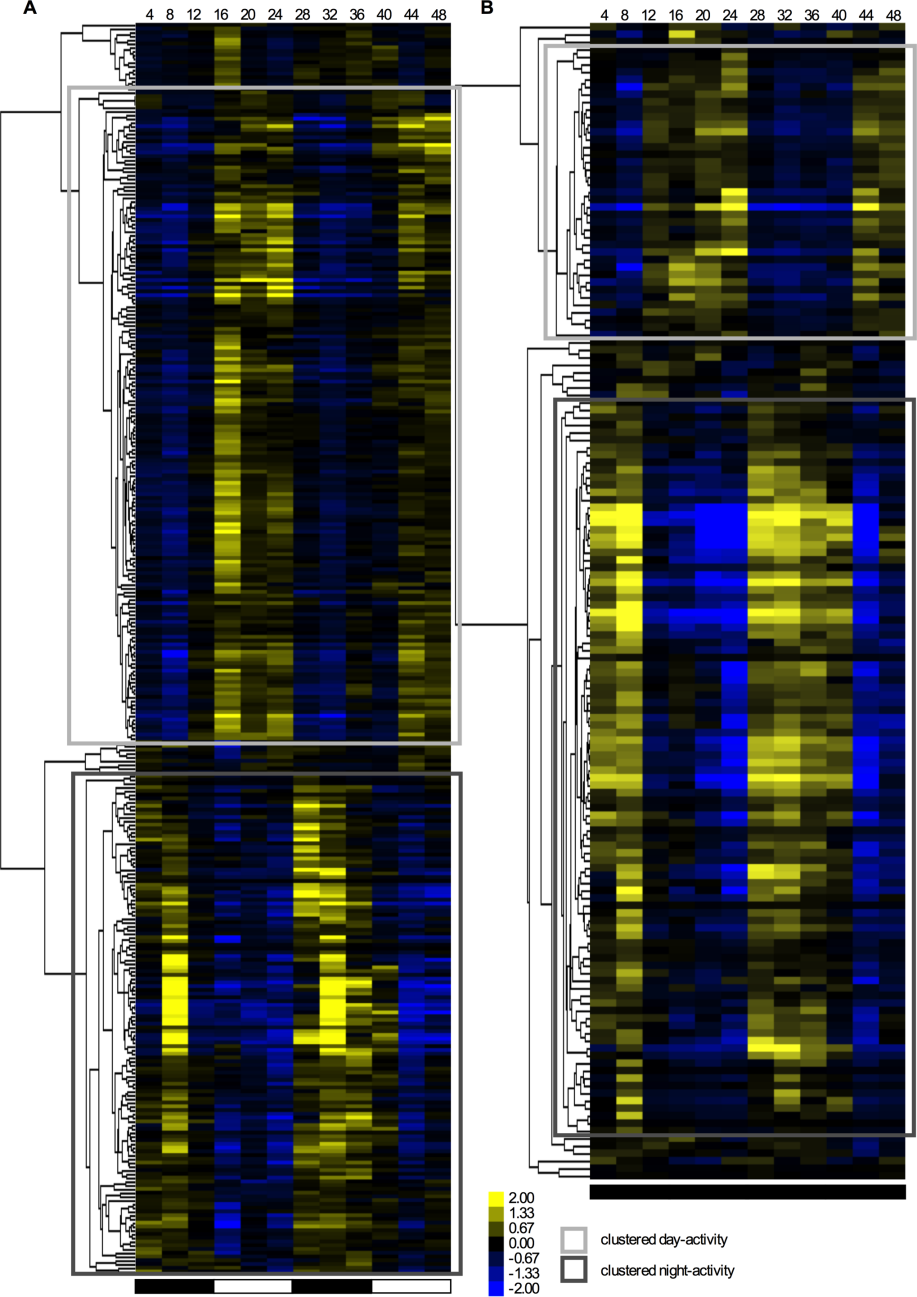
Clustering of genes with rhythmic transcripts. (A) A heat map representation of the clustering of genes found to have rhythmic transcripts based on their expression profiles over a time course of 48 h in LD. (B) A heat map representation of the clustering of genes found to have rhythmic transcripts based on their expression profiles over a time course of 48 h in DD. The squares indicate two clusters: genes showing up-regulation during the light phase (light grey squares), and genes showing up-regulation during the dark phase (dark grey squares).

**Fig 5:**
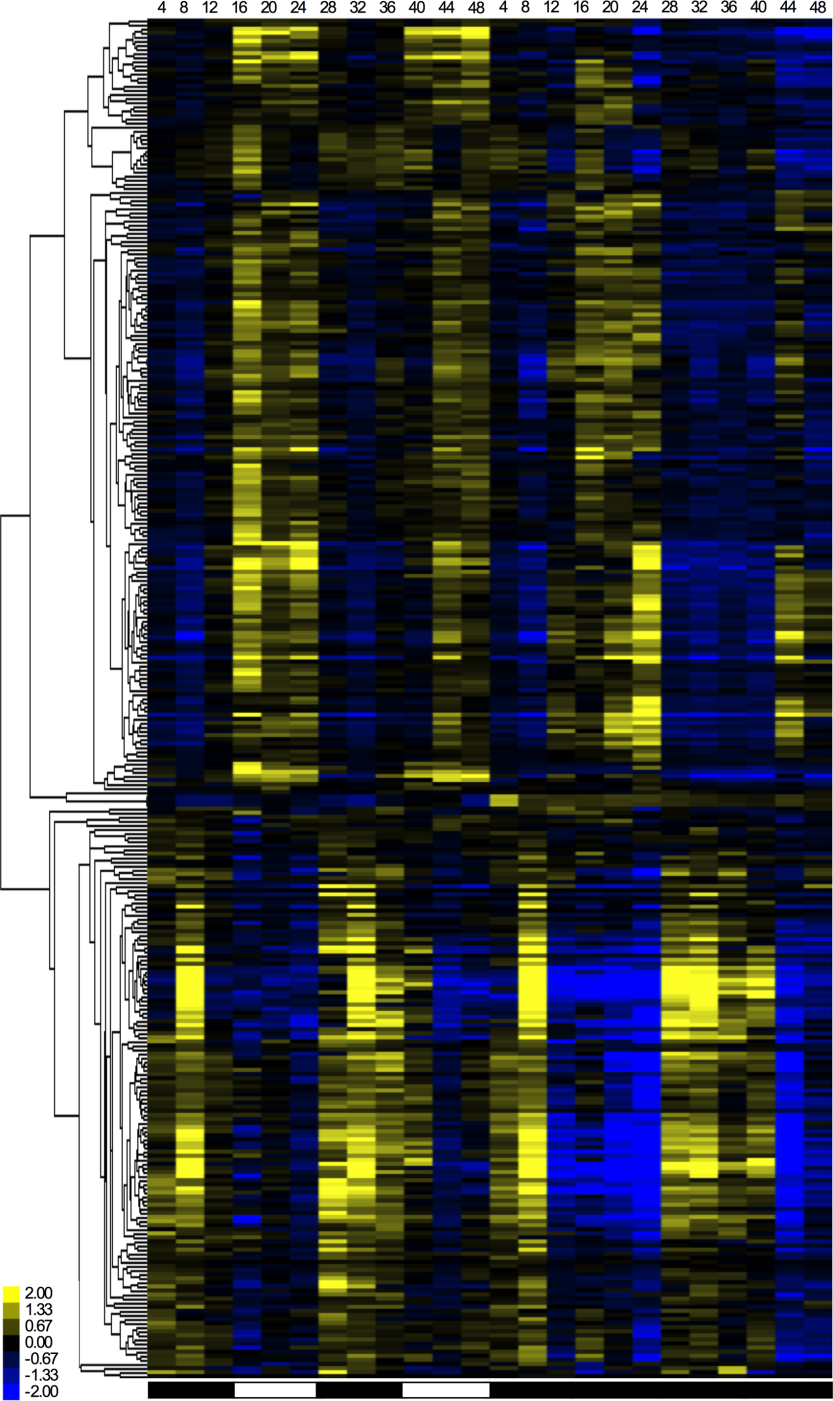
Heat map of gene expression patterns in LD and DD cultures. A heat map representation of genes found to oscillate in their expression over 48 h in LD conditions is concatenated with their expression profiles over 48 h in DD conditions.

Concentrations of expression to specific times of day have been observed in *Neurospora*, where circadian transcripts generally peak at different times depending on their function [33, 34]. Here, we performed cluster analyses on rhythmic genes in *O*. *kimflemingiae* (JTK_CYCLE at p≤0.057) to determine time-of-day (phase) expression. The majority of genes with rhythmic transcripts cluster into either the light or the dark phase (as opposed to dawn/dusk [34]; Fig 4). In LD, 52% of the cycling genes were up-regulated in the light phase and down-regulated in the dark. The opposite expression pattern was observed in 40% of the genes: up-regulation during the dark phase and down-regulation during the light phase (Fig 4A). We observed a similar pattern under DD conditions, where 25% of rhythmic genes were up-regulated during the subjective day and down-regulated during subjective night. For 64%, the peak expression was in the subjective night (Fig 4B). The majority of rhythmic genes in DD thus had peak expression during the subjective night. Indeed, when we looked at the 26 genes that are cycling in both LD and DD, nineteen (73%) of them peaked during the dark or subjective night phase. We again did another cluster analysis on all rhythmic genes found under LD conditions with concatenation of their expression levels in DD (Fig 5). The resulting heat map visualizes how rhythmicity of many genes that peaked in the light phase damp after 24 h in DD. Conversely, many genes that are up-regulated during the dark phase remained rhythmic in DD. This suggests that the majority of the rhythmic day-active genes are probably light-driven rather than endogenously rhythmic. Among the night-active genes, endogenous rhythmicity seems more likely to be found.

To obtain insight into the biological processes that are represented during the (subjective) light and dark phases, we performed enrichment analyses on the functional annotations of the genes within these clusters. Information about annotations and clustered activity for each rhythmic gene can be found in S1 and S2 Data Files. Among the genes that peak in the light phase, TFs were significantly over-represented (11 genes, corrected p-value 0.003). During the dark phase, under cycling conditions, a significant over-representation for SPs and SSPs was found (50 genes total, corrected p-values 1.13E-16 and 4E-07, respectively). In fact, they comprised 37% of all genes that peaked in the dark. Among these genes were five annotated enterotoxins with secretion signals, and four annotated proteases, as well as the above-mentioned tyrosinase, predicted to be secreted, protein tyrosine phosphatase, and homolog of *N*. *crassa ccg-6*. In DD, enrichments were only found among the genes that peaked during the subjective night. Again, SPs and SSPs were over-represented (23 genes total, corrected p-values 0.0002 and 0.0006, respectively), as well as genes encoding for secondary metabolites from annotated Cluster 8 (2 out of a total of 5 genes within the cluster, corrected p-value 0.0012). These secondary metabolites are both annotated to be involved in oxidation-reduction processes (a predicted P450 monooxygenase and a FAD dependent monooxygenase). Among the genes with secretion signals were the genes that appeared to be expressed in the (subjective) night in Fig 2. We also identified another endopeptidase, predicted to be secreted, a glycosyl hydrolase, and various SPs and SSPs with unknown function. This suggests that while a significant number of TFs are expressed during the light phase, the rhythmic *O*. *kimflemingiae* genes encoding enzymes and small bioactive compounds that may interact with the ant host are predominantly expressed during the dark phase.

## Conclusions

In this study, we have provided the first evidence that the behavior manipulating fungus *O*. *kimflemingiae* has a circadian clock. Using a culture method that allows for the performance of time course experiments in LD and in DD, we have demonstrated that *O*. *kimflemingiae* cultures exhibit daily oscillations in their mRNA levels. We identified homologs of fungal clock genes in the genome of *O*. *kimflemingiae* and confirmed their rhythmicity in LD with RNA-Seq and RT-QPCR. Among the other rhythmic, likely clock-controlled, genes (ccg’s), a significant number are predicted to be involved in parasite-host interactions. These could thus be important in establishing behavioral manipulation. Our finding that the activity patterns of these ccg’s are seemingly synchronized to a certain time of day additionally indicates that daily timing could be involved in insect infection and manipulation. Our data thus sets the stage for further detailed investigations of the involvement of biological clocks in the mechanisms of infection and manipulation. At this time, clock knock-outs can not yet be made for *O*. *kimflemingiae*. The development of the molecular tools to facilitate this will aid in the true characterization of the core clock homologs in this fungus and how they potentially regulate effector genes. This will lead to a better understanding of the pathways and timing that lead to key parasite-host interactions that are taking place. Such an understanding may eventually support development of more effective strategies for the biological control of insect pests.

## Supporting information

S1 Table Culturing conditions trials. Media compositions and culturing conditions tested to induce *O*. *kimflemingiae* blastospore growth.
(DOCX)

S1 Fig Summary of JTK_CYCLE results. (A) Venn diagram of all rhythmic candidates found in this study under LD and DD conditions. (B) Histogram visualizing the frequency of the periods found for all rhythmic candidates under LD and DD conditions. The median period under LD conditions was 24 h, while under DD conditions we found a median period of 26 h.
(TIFF)

S2 Fig Expression of candidate genes involved in circadian regulation. Expression profiles of four *O*. *kimflemingiae* clock gene homologs and *cryptochrome* over the course of 48 h under LD (A-E) and DD (F-J) conditions. Normalized expression levels from RNA-Seq and RT-QPCR data obtained from the same biological replicate (Time Course 1), and RT-QPCR data obtained from an independently obtained biological replicate (Time Course 2) have been plotted as mean Z-scores. Error bars indicate Standard Error of the Mean.
(TIFF)

S1 Data File Rhythmic genes under LD conditions. All genes found to be cycling with a likelihood of p≤0.057 (p-value *wc-1* set as threshold) under LD conditions in the 4h-interval dataset. For each gene the ProteinID is given, as well as its functional annotation, the NCBI blast result, the JTK_CYCLE output, the expression values (RPKM) of each time course sample under LD and DD conditions, and the clustered day or night-activity.
(XLSX)

S2 Data File Rhythmic genes under DD conditions. All genes found to be cycling with a likelihood of p≤0.057 under DD conditions in the 4h-interval dataset. For each gene the ProteinID is given, as well as its functional annotation, the NCBI blast result, the JTK_CYCLE output, the expression values (RPKM) of each time course sample under DD conditions, and the clustered day or night-activity.
(XLSX)
