# Supplementary material for: Daily rhythms and enrichment patterns in the transcriptome of the behavior-manipulating parasite Ophiocordyceps kimflemingiae
Source: PLoS One. 2017 Nov 3;12(11):e0187170. doi: 10.1371/journal.pone.0187170 (PMC5669440; doi:10.1371/journal.pone.0187170)
Supplement: S1 Table — Media compositions and culturing conditions tested to induce O. kimflemingiae blastospore growth. (DOCX) [file pone.0187170.s001.docx]

| **Media name** | **Components** | **Growth conditions** | **Blastospore formation?** |
| --- | --- | --- | --- |
| Grace’s + 10% FBS | Grace’s (Sigma) Fetal Bovine Serum (Gibco) | 20 mL in 25 cm^2^ tissue culture flask, 60 rpm | Yes |
|  |  | 25 mL in 100 mL Erlenmeyer flask, 60 rpm | No |
|  |  | 5 mL in 6-well plate, stationary | No |
| Grace’s + 10% FBS  + 0.4 % Tween-80[1, 2] | Grace’s (Sigma) Fetal Bovine Serum (Gibco)  Tween-80 (Sigma) | 20 mL in 25 cm^2^ tissue culture flask, 60 rpm | Yes |
|  |  | 25 mL in 100 mL Erlenmeyer flask, 60 rpm | No |
|  |  | 5 mL in 6-well plate, stationary | No |
| Grace’s + 10% FBS  + 5 % PEG200[2, 3] | Grace’s (Sigma) Fetal Bovine Serum (Gibco)  PEG200 (Sigma) | 20 mL in 25 cm^2^ tissue culture flask, 60 rpm | Yes |
|  |  | 25 mL in 100 mL Erlenmeyer flask, 60 rpm | No |
|  |  | 5 mL in 6-well plate, stationary | No |
| GYS[4] with 2.7% glucose | As listed in Kocharin and Wongsa, 2006 | 20 mL in 25 cm^2^ tissue culture flask, 60 rpm | No |
|  |  | 25 mL in 100 mL Erlenmeyer flask, 60 rpm | No |
|  |  | 5 mL in 6-well plate, stationary | No |
| GYS[4] with 2.7% glucose  + 0.4 % Tween-80 | As listed in Kocharin and Wongsa, 2006  + Tween-80 (Sigma) | 20 mL in 25 cm^2^ tissue culture flask, 60 rpm | No |
|  |  | 25 mL in 100 mL Erlenmeyer flask, 60 rpm | No |
|  |  | 5 mL in 6-well plate, stationary | No |
| GYS[4] with 2.7% glucose  + 5 % PEG200 | As listed in Kocharin and Wongsa, 2006  + PEG200 (Sigma) | 20 mL in 25 cm^2^ tissue culture flask, 60 rpm | No |
|  |  | 25 mL in 100 mL Erlenmeyer flask, 60 rpm | No |
|  |  | 5 mL in 6-well plate, stationary | No |
| SDB | Sabourad Broth (Sigma) | 20 mL in 25 cm^2^ tissue culture flask, 60 rpm | No |
|  |  | 25 mL in 100 mL Erlenmeyer flask, 60 rpm | No |
|  |  | 5 mL in 6-well plate, stationary | No |
| SDB  + 0.4 % Tween-80 | Sabourad Broth (Sigma)  Tween-80 (Sigma) | 20 mL in 25 cm^2^ tissue culture flask, 60 rpm | No |
|  |  | 25 mL in 100 mL Erlenmeyer flask, 60 rpm | No |
|  |  | 5 mL in 6-well plate, stationary | No |
| SDB  + 5 % PEG200 | Sabourad Broth (Sigma)  PEG200 (Sigma) | 20 mL in 25 cm^2^ tissue culture flask, 60 rpm | No |
|  |  | 25 mL in 100 mL Erlenmeyer flask, 60 rpm | No |
|  |  | 5 mL in 6-well plate, stationary | No |
| GM medium[5] | As listed in Ying and Feng, 2006 | 20 mL in 25 cm^2^ tissue culture flask, 60 rpm | No |
|  |  | 25 mL in 100 mL Erlenmeyer flask, 60 rpm | No |
|  |  | 5 mL in 6-well plate, stationary | No |
| GM medium[5]  + 0.4 % Tween-80 | As listed in Ying and Feng, 2006 + Tween-80 (Sigma) | 20 mL in 25 cm^2^ tissue culture flask, 60 rpm | No |
|  |  | 25 mL in 100 mL Erlenmeyer flask, 60 rpm | No |
|  |  | 5 mL in 6-well plate, stationary | No |
| GM medium[5]  + 5 % PEG200 | As listed in Ying and Feng, 2006 + PEG200 (Sigma) | 20 mL in 25 cm^2^ tissue culture flask, 60 rpm | No |
|  |  | 25 mL in 100 mL Erlenmeyer flask, 60 rpm | No |
|  |  | 5 mL in 6-well plate, stationary | No |
| Adamek’s[6] | As listed in Adamek, 1963 | 20 mL in 25 cm^2^ tissue culture flask, 60 rpm | No |
|  |  | 25 mL in 100 mL Erlenmeyer flask, 60 rpm | No |
|  |  | 5 mL in 6-well plate, stationary | No |
| Adamek’s[6]  + 0.4 % Tween-80 | As listed in Adamek, 1963 + Tween-80 (Sigma) | 20 mL in 25 cm^2^ tissue culture flask, 60 rpm | No |
|  |  | 25 mL in 100 mL Erlenmeyer flask, 60 rpm | No |
|  |  | 5 mL in 6-well plate, stationary | No |
| Adamek’s[6]  + 5 % PEG200 | As listed in Adamek, 1963 + PEG200 (Sigma) | 20 mL in 25 cm^2^ tissue culture flask, 60 rpm | No |
|  |  | 25 mL in 100 mL Erlenmeyer flask, 60 rpm | No |
|  |  | 5 mL in 6-well plate, stationary | No |

1. Fargues J, Smits N, Vidal C, Vey A, Vega F, Mercadier G, et al. Effect of liquid culture media on morphology, growth, propagule production, and pathogenic activity of the Hyphomycete, *Metarhizium flavoviride*. Mycopathologia. 2002;154(3):127-38. doi: Doi 10.1023/A:1016068102003.

2. Kleespies RG, Zimmermann G. Production of blastospores by three strains of *Metarhizium anisopliae* (metch.) sorokin in submerged culture. Biocontrol Science and Technology. 1992;2(2):127-35. doi: dx.doi.org/10.1080/09583159209355226.

3. Humphreys AM, Matewele P, Trinci APJ, Gillespie AT. Effects of water activity on morphology, growth and blastospore production of *Metarhizium* *anisopliae*, *Beauveria bassiana* and *Paecilomyces farinosus* in batch and fed-batch culture. Mycol Res. 1989;92:257-64.

4. Kocharin K, Wongsa P. Semi-defined medium for in vitro cultivation of the fastidious insect pathogenic fungus *Cordyceps unilateralis*. Mycopathologia. 2006;161(4):255-60. doi: 10.1007/s11046-005-0224-x.

5. Ying SH, Feng MG. Novel blastospore-based transformation system for integration of phosphinothricin resistance and green fluorescence protein genes into *Beauveria bassiana*. Appl Microbiol Biotechnol. 2006;72(1):206-10. doi: 10.1007/s00253-006-0447-x.

6. Adamek L. Submerse cultivation of the fungus *Metarrhizium anisopliae* (Metsch.). Folia Microbiol (Praha). 1965;10:255-7.
