# Supplementary figures and images for: Daily rhythms and enrichment patterns in the transcriptome of the behavior-manipulating parasite Ophiocordyceps kimflemingiae
Source: PLoS One. 2017 Nov 3;12(11):e0187170. doi: 10.1371/journal.pone.0187170 (PMC5669440; doi:10.1371/journal.pone.0187170)

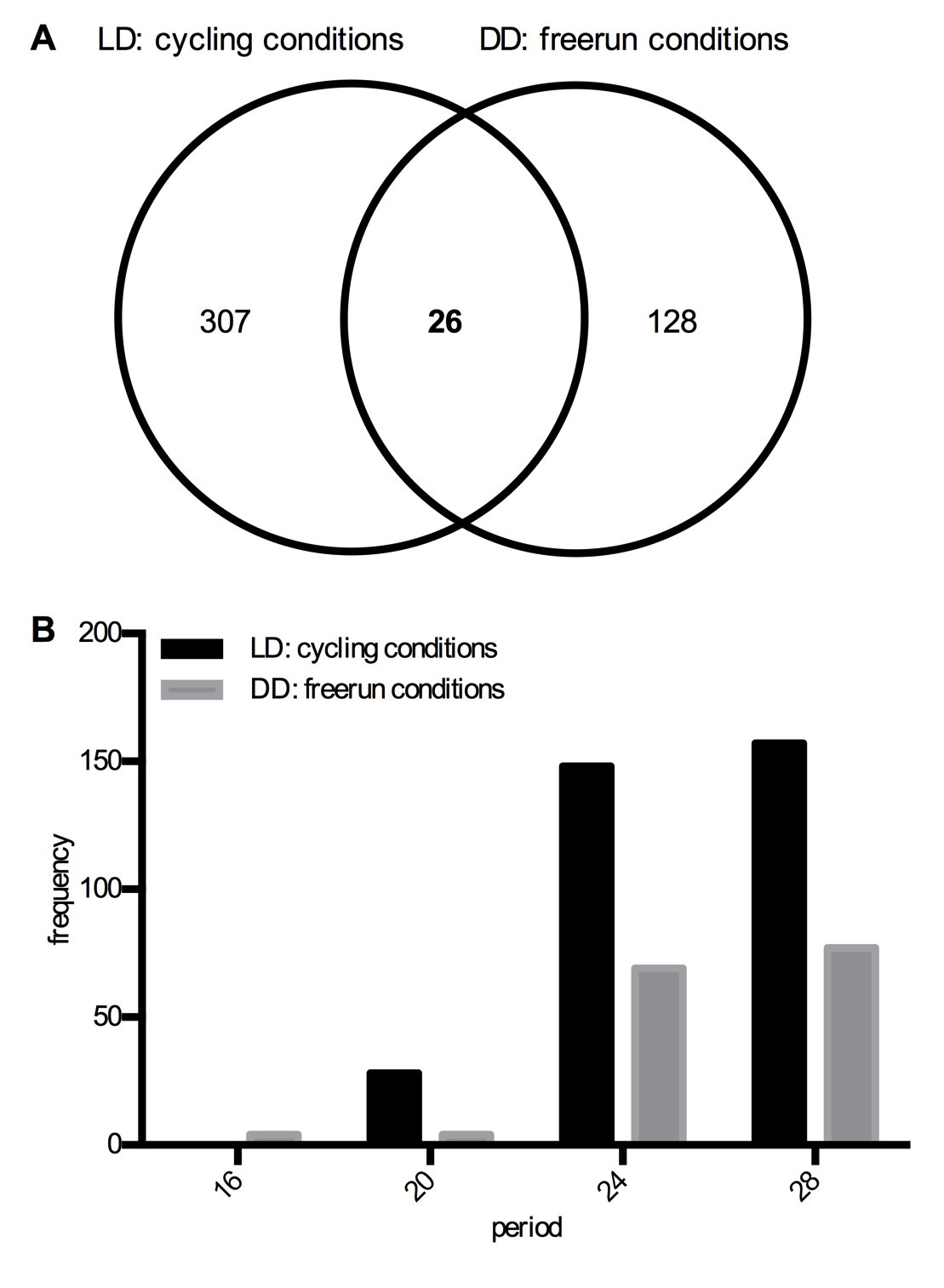

Supplement: S1 Fig — (A) Venn diagram of all rhythmic candidates found in this study under LD and DD conditions. (B) Histogram visualizing the frequency of the periods found for all rhythmic candidates under LD and DD conditions. The median period under LD conditions was 24 h, while under DD conditions we found a median period of 26 h. (TIFF) [file pone.0187170.s002.tiff]

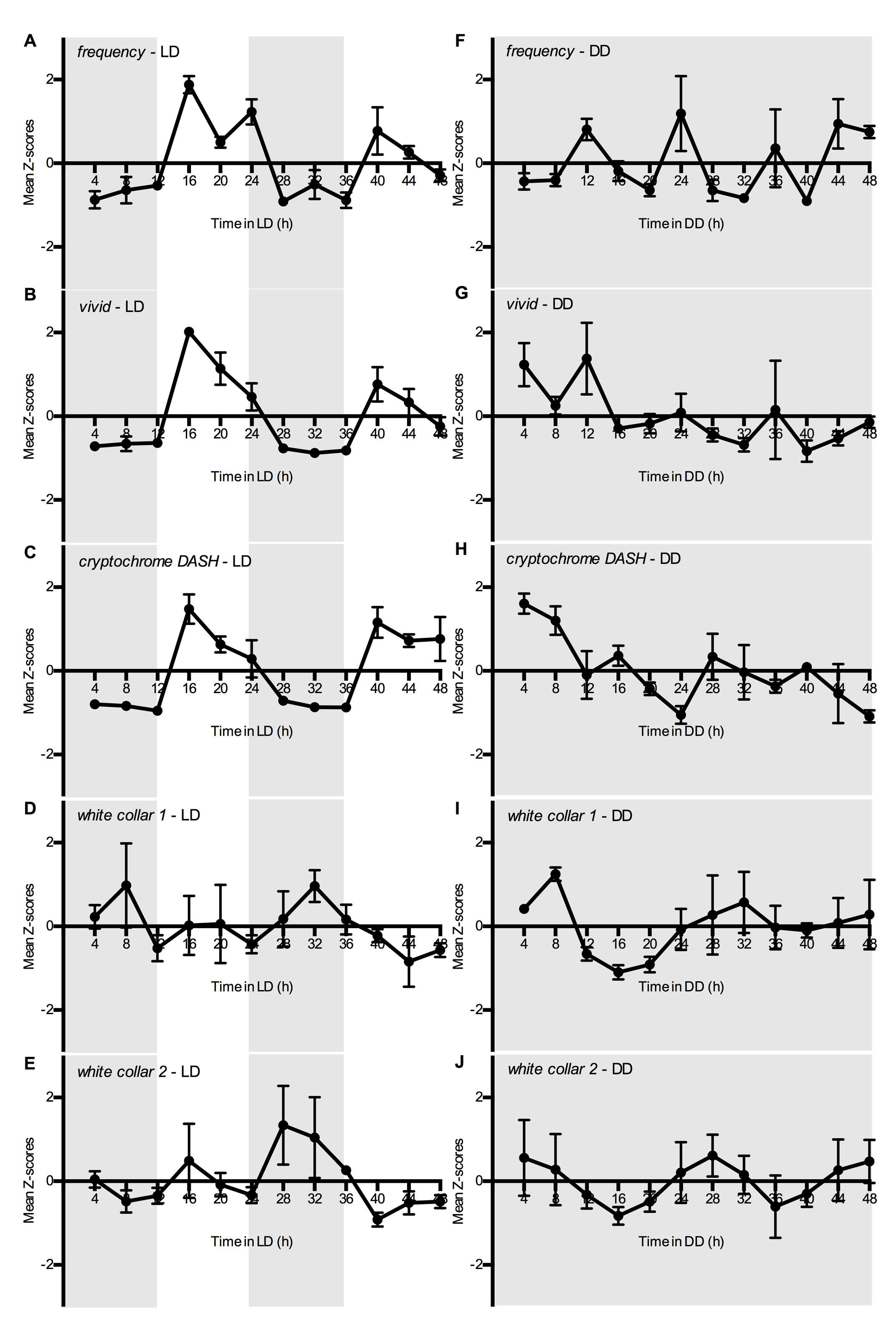

Supplement: S2 Fig — Expression profiles of four O. kimflemingiae clock gene homologs and cryptochrome over the course of 48 h under LD (A-E) and DD (F-J) conditions. Normalized expression levels from RNA-Seq and RT-QPCR data obtained from the same biological replicate (Time Course 1), and RT-QPCR data obtained from an independently obtained biological replicate (Time Course 2) have been plotted as mean Z-scores. Error bars indicate Standard Error of the Mean. (TIFF) [file pone.0187170.s003.tiff]
